# Antidepressant-Like Effects of Pithecellobium dulce Fruit Extract in a Chronic Unpredictable Stress-Induced Zebrafish Larvae Model: Behavioral, Molecular, and Phytochemical Insights

**DOI:** 10.7759/cureus.107622

**Published:** 2026-04-23

**Authors:** Priyadharshini Vellore Sureshkumar, Ramesh H S, Satishkumar Vadlamudi, Usha Sadasivan

**Affiliations:** 1 Dr APJ Abdul Kalam Center of Excellence in Innovation and Entrepreneurship, Dr. M.G.R. Educational and Research Institute, Chennai, IND; 2 Pharmacology, Sri Chamundeshwari Medical College, Channapatna, IND; 3 Pharmacology, Sri Lalithambigai Medical College and Hospital, Chennai, IND

**Keywords:** brain-derived neurotrophic factor (bdnf), neurodegenerative diseases and depression, pithecellobium dulce, tph2, zebrafish model

## Abstract

Background

Depression is a multifactorial neuropsychiatric disorder involving alterations in neuroplasticity, neurotransmission, and oxidative stress. Zebrafish larvae are a useful model for studying stress-induced behavioral changes. *Pithecellobium dulce*, rich in bioactive phytochemicals, has reported antioxidant properties, but its effects in stress models remain underexplored.

Methods

Zebrafish larvae were subjected to a chronic unpredictable stress (CUS) paradigm and divided into control, CUS, extract, CUS + standard (sertraline), and CUS + *P. dulce* extract (1 mg/mL) groups (n = 20). Behavioral assays included color preference, vertical exploration, thigmotaxis, and light-dark tests. Toxicity assessment, phytochemical screening, gas chromatography-mass spectrometry (GC-MS) analysis, DPPH antioxidant assay, and quantitative real-time PCR analysis of brain-derived neurotrophic factor (BDNF) and tryptophan hydroxylase-2 (TPH2) were performed.

Results

CUS induced significant behavioral deficits, including reduced exploration and increased dark preference. *Pithecellobium dulce* treatment improved these behaviors without observable toxicity at 1 mg/mL (no observed adverse effect concentration). Phytochemical analysis confirmed alkaloids, flavonoids, tannins, saponins, and phenolics. GC-MS identified bioactive compounds such as 5-hydroxymethylfurfural. The extract showed concentration-dependent antioxidant activity (max 84.83%; IC_50_ ~38 µg/mL). BDNF and TPH2 expression were reduced in CUS and showed partial restoration following treatment.

Conclusions

*Pithecellobium dulce* extract was associated with improvement in stress-induced behavioral alterations, potentially involving antioxidant effects and modulation of neuroplasticity and serotonergic pathways. However, findings are based on indirect evidence and require further mechanistic and translational validation.

## Introduction

Depression is a leading cause of global health-related disability and significantly impairs psychosocial functioning and quality of life. It has been projected to become one of the foremost contributors to the global disease burden worldwide [1]. Epidemiological evidence indicates that depressive disorders often begin early in life, with a substantial burden observed among children and adolescents. A recent systematic review and meta-analysis reported that approximately one in five children and adolescents worldwide experience depressive symptoms, with increasing prevalence over time [2]. Similarly, another global analysis found that a significant proportion of adolescents exhibit elevated depressive symptoms, highlighting the urgent need for early identification and effective therapeutic strategies [3].

The pathophysiology of depression has been extensively linked to the dysregulation of monoaminergic neurotransmission. The classical monoamine hypothesis proposes that depression arises from deficiencies in central serotonergic and noradrenergic systems [4]. Serotonin biosynthesis is critically regulated by tryptophan hydroxylase-2 (TPH2), the rate-limiting enzyme in central serotonin production, and alterations in TPH2 activity have been associated with depression-like behaviors and changes in serotonin metabolism across animal models [5]. However, this hypothesis alone does not fully explain the complexity of depressive disorders. Emerging evidence highlights the role of impaired neuroplasticity, particularly reduced expression of brain-derived neurotrophic factor (BDNF), which is essential for neuronal survival, synaptic plasticity, and neurogenesis [6]. Additionally, neuroinflammation has been implicated in depression, with activated microglia releasing pro-inflammatory cytokines that contribute to neuronal dysfunction [7].

Despite advances in pharmacological treatments, current antidepressant therapies are associated with several limitations. Selective serotonin reuptake inhibitors (SSRIs) and related drugs are often accompanied by adverse effects such as insomnia, gastrointestinal disturbances, and weight changes, which may affect patient compliance [8]. Furthermore, a substantial proportion of patients fail to respond adequately to first-line treatments, leading to treatment-resistant depression [9]. These limitations underscore the need for safer, multi-target, and more effective therapeutic alternatives.

In recent years, plant-derived compounds have gained attention as potential therapeutic agents for depression due to their multi-target mechanisms and improved safety profiles. Phytochemicals such as flavonoids have demonstrated antidepressant-like effects by modulating monoaminergic pathways, neuroinflammation, and neuroplasticity [10]. Additionally, plant-derived polyphenols have been shown to influence the gut-brain axis and regulate neuroinflammatory processes [11]. *Pithecellobium dulce*, a medicinal plant widely used in traditional systems of medicine, contains bioactive compounds such as flavonoids, tannins, and alkaloids and exhibits antioxidant and neuroprotective properties [12]. However, its antidepressant potential and underlying molecular mechanisms remain underexplored.

Zebrafish (Danio rerio) have emerged as a robust and translationally relevant model for studying neuropsychiatric disorders due to their genetic and neurobiological similarity to humans. Zebrafish larvae represent a valuable model for studying early-life stress responses due to their rapid neurodevelopment, optical transparency, and suitability for high-throughput behavioral and molecular analyses. Early-life stages are particularly relevant, as depression and stress-related disorders often emerge during childhood and adolescence. Furthermore, these models exhibit conserved neurotransmitter systems, including serotonergic and neurotrophic pathways, along with well-characterized behavioral paradigms relevant to anxiety and depression, making them appropriate for investigating the effects of stress and therapeutic interventions [13,14]. Chronic unpredictable stress (CUS) paradigms reliably induce anxiety- and depression-like behaviors in zebrafish, mimicking mammalian stress responses [15]. Moreover, plant-derived compounds have been shown to reverse stress-induced behavioral alterations in zebrafish models [16].

Despite these advances, the antidepressant potential of *P. dulce* has not been explored in zebrafish models. Therefore, this study aimed to evaluate stress-induced behavioral alterations in zebrafish larvae and associated molecular changes in neurotrophic (BDNF) and serotonergic (TPH2) gene expression, along with the characterization of the phytochemical composition and antioxidant potential of *P. dulce* fruit extract. We hypothesized that CUS would induce behavioral impairments and downregulate BDNF and TPH2 expression, whereas treatment with *P. dulce* extract would mitigate these effects and partially restore behavioral and molecular parameters toward control levels.

## Materials and methods

Study design

This experimental study was conducted to evaluate the antidepressant-like effects and safety profile of *P. dulce* fruit extract in a CUS-induced zebrafish larvae model. Behavioral assays were performed to assess anxiety- and depression-like phenotypes. These were followed by toxicological and molecular analyses.

Zebrafish husbandry and maintenance

Wild-type zebrafish (Danio rerio) were maintained under standard laboratory conditions in a recirculating aquatic system at 28 ± 1°C under a 14:10-hour light-dark cycle [17]. Fish were fed twice daily with a commercial diet.

Larval selection and stress induction

Zebrafish larvae were subjected to a CUS paradigm involving randomly applied stressors such as social isolation, temperature variation, feed timing alterations, predator cues, tank tapping, tank tilting, overcrowding, repeated tank transfer, light-dark alterations, feed delay, feed deprivation, tank tapping, and aeration stress. Five stressors were applied per day for 14 days in a randomized sequence to prevent habituation [15]. Only morphologically normal and actively swimming larvae were included in the study (Table 1).

**Table 1:** Standardized 14-day CUS paradigm in zebrafish larvae Zebrafish larvae were subjected to a 14-day CUS protocol consisting of five stressors applied daily in a randomized sequence to prevent habituation. Each stressor was administered with a defined duration and intensity, as indicated. Stressors included social isolation, temperature variation, predator cues, tank manipulation, aeration stress, feeding alterations, and light–dark cycle disruptions. This standardized protocol was designed to induce depression-like behavioral phenotypes. The order of stressors was varied across days to minimize predictability and stress adaptation. CUS, chronic unpredictable stress; °C, degree Celsius; h, hours; min, minutes

Day	Stressor 1	Stressor 2	Stressor 3	Stressor 4	Stressor 5
1	Social isolation (2 h)	Temperature ↓ to 24°C (1 h)	Tank tilting (30°, 30 min)	Light/dark alteration (1 h)	Feed delay (6 h)
2	Overcrowding (2× density, 2 h)	Predator cue exposure (visual, 30 min)	Tank transfer (6 times)	Aeration stress (low O₂, 30 min)	Light exposure (continuous, 1 h)
3	Temperature ↑ to 32°C (1 h)	Social isolation (2 h)	Tank tapping disturbance (10 min)	Feed deprivation (8 h)	Dark exposure (1 h)
4	Tank tilting (30°, 30 min)	Predator cue (olfactory, 30 min)	Overcrowding (2 h)	Temperature ↓ to 24°C (1 h)	Light/dark reversal (1 h)
5	Tank transfer (6 times)	Social isolation (2 h)	Aeration stress (30 min)	Feed delay (6 h)	Continuous light (1 h)
6	Overcrowding (2 h)	Temperature ↑ to 32°C (1 h)	Predator cue (visual, 30 min)	Tank tapping (10 min)	Dark exposure (1 h)
7	Light/dark alteration (1 h)	Social isolation (2 h)	Tank tilting (30°, 30 min)	Feed deprivation (8 h)	Aeration stress (30 min)
8	Tank transfer (6 times)	Predator cue (olfactory, 30 min)	Temperature ↓ to 24°C (1 h)	Overcrowding (2 h)	Continuous light (1 h)
9	Social isolation (2 h)	Tank tapping (10 min)	Temperature ↑ to 32°C (1 h)	Feed delay (6 h)	Dark exposure (1 h)
10	Predator cue (visual, 30 min)	Tank tilting (30°, 30 min)	Overcrowding (2 h)	Aeration stress (30 min)	Light/dark reversal (1 h)
11	Tank transfer (6 times)	Social isolation (2 h)	Temperature ↓ to 24°C (1 h)	Feed deprivation (8 h)	Continuous light (1 h)
12	Overcrowding (2 h)	Predator cue (olfactory, 30 min)	Tank tapping (10 min)	Aeration stress (30 min)	Dark exposure (1 h)
13	Temperature ↑ to 32°C (1 h)	Social isolation (2 h)	Tank tilting (30°, 30 min)	Feed delay (6 h)	Light/dark alteration (1 h)
14	Tank transfer (6 times)	Predator cue (visual, 30 min)	Overcrowding (2 h)	Temperature ↓ to 24°C (1 h)	Continuous light (1 h)

Ethical approval

All experimental procedures were conducted in accordance with the Committee for the Purpose of Control and Supervision of Experiments on Animals (CPCSEA) guidelines and approved by the Institutional Animal Ethics Committee (IAEC) of ACS Medical College and Hospital, Chennai, Tamil Nadu, India. Sample sizes were determined based on standard zebrafish experimental practices and IAEC recommendations to minimize animal use while maintaining experimental reliability.

Preparation of *P. dulce* extract

Fresh fruits of *P. dulce* were washed, air-dried at room temperature, and ground into a fine powder. Approximately 70 g of the dried powder was subjected to ethanolic extraction using a 1:10 (w/v) solvent ratio (70 g in 700-mL ethanol). The mixture was initially sonicated for 15 min to enhance solvent penetration, followed by incubation in a shaking incubator for 12 hours to facilitate extraction. The extract was then filtered using Whatman filter paper, and the solvent was removed under reduced pressure using a rotary evaporator to obtain a concentrated extract. The dried extract was stored at 4°C in airtight amber-colored vials until further use.

For experimental applications, the extract was reconstituted in ethanol to prepare a stock solution, and a working concentration of 1 mg/mL was prepared in embryo medium [18]. The extraction yield was approximately 10% (w/w) based on the initial dry weight of the plant material.

Embryo medium preparation

E3 embryo medium was prepared according to standard protocols. A 60× stock solution was prepared by dissolving sodium chloride (NaCl, 34.8 g), potassium chloride (KCl, 1.6 g), calcium chloride dihydrate (CaCl₂·2H₂O, 5.8 g), and magnesium chloride hexahydrate (MgCl₂·6H₂O, 9.78 g) in 1.95 L of double-distilled water (ddH₂O). The pH was adjusted to 7.2 using sodium hydroxide (NaOH) or hydrochloric acid (HCl), and the final volume was made up to 2 L with ddH₂O.

The working solution (1× E3) was prepared by diluting 16.5 mL of the 60× stock in 1 L of ddH₂O, resulting in final concentrations of sodium chloride (5.0 mM), potassium chloride (0.17 mM), calcium chloride (0.33 mM), and magnesium chloride (0.33 mM). Methylene blue (100 µL of 1% solution) was added to prevent fungal contamination. Embryos were maintained in 1× E3 embryo medium throughout the study unless otherwise specified.

Phytochemical screening

Preliminary qualitative phytochemical analysis was performed using standard methods. Alkaloids were detected using Dragendorff’s reagent, flavonoids using concentrated HCl, tannins using ferric chloride, saponins by foam test, and phenolic compounds using Folin-Ciocalteu reagent [18].

Gas chromatography-mass spectrometry analysis

Phytochemical characterization of the extract was carried out using gas chromatography-mass spectrometry (GC-MS) with an HP-5 MS capillary column. Helium was used as the carrier gas at a constant flow rate of 1 mL/min. The injector temperature was maintained at 250°C, and the oven temperature was programmed from 60°C to 280°C. Compounds were identified by comparison with the National Institute of Standards and Technology (NIST) library database [19].

Antioxidant activity (DPPH assay)

The antioxidant activity of the extract was assessed using the DPPH free radical scavenging assay. Different concentrations of the plant extract (10-80 µg/mL) were prepared and adjusted to a final volume of 1 mL using dimethyl sulfoxide or ethanol. To each sample, 2.5 mL of 0.1-mM DPPH solution was added. A control was prepared using 3 mL of DPPH solution without the extract. The reaction mixtures were incubated in the dark for 20 min, and the absorbance was measured at 517 nm using a spectrophotometer [20].

The percentage radical scavenging activity (%RSA) was calculated using the following formula:

%RSA = [(Absorbance of Control − Absorbance of Sample) / Absorbance of Control] × 100.

The IC₅₀ value was estimated from the concentration-response curve as the concentration required to achieve 50% radical scavenging activity. All measurements were performed in triplicate (n = 3), and mean values were used for analysis.

Experimental groups

Zebrafish larvae were randomly assigned to five experimental groups (n = 20 per group):

Control group: larvae maintained under standard conditions without exposure to stress or treatment.

CUS group: larvae subjected to CUS without any treatment.

Extract group: larvae treated with *P. dulce* extract (1 mg/mL) without exposure to stress to evaluate baseline effects and safety. The selected concentration (1 mg/mL) was based on preliminary toxicity screening and literature reports to ensure non-lethal exposure.

CUS + standard group: larvae exposed to CUS and treated with sertraline hydrochloride (10 µM), and an SSRI was used as a reference antidepressant.

CUS + extract group: larvae exposed to CUS and treated with *P. dulce* extract (1 mg/mL).

Treatment regimen

Zebrafish larvae were exposed to *P. dulce* extract and the standard drug sertraline hydrochloride using a gradual dose-escalation approach to minimize acute stress. For *P. dulce* extract, larvae were exposed on day 1 to 0.25 mg/mL (25% of the final concentration) for 6 h, followed by 0.5 mg/mL for the next 6 h, and 0.75 mg/mL for an additional 6 h, after which the full target concentration of 1 mg/mL was achieved within 24 h. Similarly, for sertraline hydrochloride, larvae were exposed to 2.5 µM (25% of the final concentration) for 6 h, followed by 5 µM for the next 6 h, and 7.5 µM for an additional 6 h, reaching the full target concentration of 10 µM within 24 h. From day 2 onwards, larvae were maintained continuously at their respective final concentrations (1 mg/mL for *P. dulce* extract and 10 µM for sertraline) for 14 days, with daily renewal of the treatment solutions to ensure stability and consistency of exposure. Control groups were maintained under identical conditions without treatment. Treatment exposure was maintained concurrently with the CUS protocol throughout the 14-day period.

Acute toxicity assay

Acute toxicity assessment was performed using zebrafish embryos in accordance with the Organisation for Economic Co-operation and Development (OECD) Guideline 236 [21]. Fertilized embryos were exposed to graded concentrations of *P. dulce *extract (1-5 mg/mL), along with a control group (0 mg/mL), and maintained up to 96 hours post-fertilization (hpf). Each treatment group consisted of 20 embryos (n = 20 per concentration).

Mortality, hatching rate, morphological abnormalities (including edema, hemorrhage, and tail detachment), and cardiac activity were monitored at 24, 48, 72, and 96 hpf. Mortality was defined based on standard lethal endpoints, including coagulation, absence of heartbeat, failure of somite formation, and non-detachment of the tail, as per OECD guidelines. The percentage mortality was calculated using the following formula:

Mortality (%) = (Number of embryos exhibiting any lethal endpoint ÷ Total number of embryos) × 100.

For dose-response analysis, survival percentages at 96 hpf were used to estimate the median lethal concentration (LC_50_) and corresponding 95% confidence interval (CI) using a generalized linear model (binomial family, logit link) implemented in R (R Foundation for Statistical Computing, Vienna, Austria). The no observed adverse effect concentration (NOAEC) and lowest observed adverse effect concentration (LOAEC) were determined based on the absence or initial presence of mortality and/or observable sublethal effects [22]. The toxicity assay was performed as a single independent experiment with n = 20 embryos per concentration.

Behavioral assessment and semi-quantitative scoring

Behavioral analyses were performed using zebrafish larvae (6-7 days post-fertilization) to evaluate stress-induced and treatment-associated changes. Each experimental group consisted of three independent biological replicates, with 20 larvae per replicate (total of 60 larvae per group). Assays included vertical exploration (tank test), locomotor and zone exploration, color preference, and light-dark preference (scototaxis) tests [23-25].

To enable integrated comparison across multiple behavioral endpoints, a semi-quantitative scoring system (0-3) was applied. Behavioral responses were converted into ordinal scores ranging from 0 to 3, where 0 = severely impaired behavior, 1 = reduced behavior, 2 = moderate response, 3 = normal behavior. For vertical exploration, scores were assigned as follows: 0 = severely impaired (no movement beyond 10 mL mark), 1 = reduced behavior (movement up to ~200 mL), 2 = moderate response (movement up to ~500 mL), and 3 = normal behavior (reaching ~700 mL in the 1,000-mL column). For locomotor and zone exploration, 0 = severely impaired (restricted to outer zone), 1 = limited movement (occasional entry to middle zone), 2 = moderate activity (frequent movement to middle zone), and 3 = normal exploration (active movement in middle and inner zones). In the color preference test, 0 = severe impairment (static preference for black), 1 = reduced response (movement between black and brown), 2 = moderate response (preference for darker colors such as green/blue), and 3 = normal behavior (preference for lighter colors such as orange/yellow with active movement). In the light-dark (black-white) test, 0 = severely impaired (remained in the black zone without movement), 1 = reduced behavior (movement within the black zone near closer to the white zone), 2 = moderate response (predominantly in the black zone with moderate entries into the white zone), and 3 = normal behavior (preference for white zone).

Behavioral scoring was performed by a single blinded observer to ensure consistency and minimize inter-observer variability. For statistical analysis, the unit of analysis was the replicate mean (n = 3), with individual larvae averaged within each replicate to avoid pseudoreplication. Multidimensional behavioral parameters were visualized using radar plots to facilitate comparison across groups. For molecular analysis, a subset of 20 larvae per group was used for RNA extraction, while the remaining animals were allowed to recover and returned to the zebrafish facility.

RNA isolation and complementary DNA synthesis

Total RNA was extracted from pooled zebrafish larvae (n = 20 per group) using a commercial RNA isolation kit (Aura Pure Tissue RNA Isolation Kit, Aura Biotech, Chennai, Tamil Nadu, India; Catalog No.: ABT-141S) according to the manufacturer’s instructions. RNA concentration and purity were assessed using a NanoDrop spectrophotometer (Agilent Technologies, Santa Clara, CA, USA), and integrity was verified by agarose gel electrophoresis. Complementary DNA (cDNA) was synthesized from the extracted RNA using a 1st Strand cDNA Synthesis Kit (Aura Biotech, Catalog No.: ABT-046S) following the manufacturer’s protocol.

Quantitative real-time PCR

Quantitative real-time PCR (qPCR) was performed using SYBR Green® chemistry. Amplification was carried out with an initial denaturation at 95°C for 120 s, followed by 35 cycles of denaturation at 95°C for 10 s and annealing/extension at 60°C for 30 s, with fluorescence acquisition at each step. A melt curve analysis was performed from 50°C to 99°C (initial hold 90 s, followed by 5-s incremental steps) to confirm amplification specificity. Primer efficiency was determined using a standard curve generated from 10-fold serial dilutions of pooled cDNA. Amplification efficiency (E) was calculated using E (%) = (10^(-1/slope)^ − 1) × 100, and only primers with efficiencies between 90-110% and correlation coefficients (R² ≥ 0.99) were used.

Relative gene expression was calculated using the 2^−ΔΔCt method [26], with β-actin as the internal housekeeping control. Gene-specific primers for β-actin, BDNF, and TPH2 were designed using Primer3 software and validated against previously published sequences. All primers were commercially synthesized (lyophilized, desalting grade), and their sequences are listed in Table 2. Each experimental group consisted of pooled biological samples, and qPCR reactions were performed in duplicate (technical replicates). Due to the use of pooled samples without independent biological replicates, gene expression results were interpreted as descriptive trends.

**Table 2:** Primer sequences used for quantitative real-time PCR analysis Length (bp), primer length in base pairs; %GC, percentage of guanine and cytosine content; Tm (°C), melting temperature of the primer

Gene	Primer Direction	Sequence (5′–3′)	Length (bp)	%GC	Tm (°C)
β-actin	Forward	CGAGCTGTCTTCCCATCCA	19	57.9	53.2
	Reverse	TCACCAACGTAGCTGTCTTTCTG	23	47.8	55.3
Bdnf	Forward	TGCGAGTTATAGTGCCGCTT	20	50.0	51.8
	Reverse	AGCCGCCGTTTACTTTTCTC	20	55.0	53.8
Tph2	Forward	TCTACTACAACCCTTACACGCAGA	24	45.8	55.7
	Reverse	CGTCACAGACGGTGGTTTAAG	20	55.0	53.8

Statistical analysis

Behavioral data are presented as mean ± standard deviation (SD) of three independent biological replicates (n = 3). Given the use of replicate-level aggregation, statistical outcomes are interpreted as indicative of group-level trends rather than precise estimates of effect size and were analyzed using Jamovi software (version 2.3.28). Normality and homogeneity of variance were assessed using the Shapiro-Wilk and Levene’s tests, respectively. Parametric data were analyzed using one-way analysis of variance (ANOVA), followed by Tukey’s post hoc test, while non-parametric data were analyzed using the Kruskal-Wallis test, followed by Dunn’s multiple comparison test with Bonferroni correction. A p-value of <0.05 was considered statistically significant.

Gene expression data were derived from pooled samples with duplicate technical measurements (n = 2 per group). Due to the absence of independent biological replicates, statistical significance testing was not performed, and results are presented descriptively as mean values.

Data visualization

Graphical representations, including DPPH line graphs, gene expression heatmaps, survival plots, and radar plots, were generated using R statistical software (version 4.5.1) with the ggplot2 package.

## Results

Phytochemical analysis of the *P. dulce* extract

Qualitative phytochemical screening of the plant extract indicated the presence of multiple bioactive secondary metabolites. The alkaloid test showed the formation of an orange-reddish precipitate, indicating a positive result. Flavonoid analysis demonstrated a distinct pink coloration upon the addition of concentrated HCl, indicating their presence. The tannin test exhibited a greenish-black coloration with ferric chloride, suggesting the presence of tannins. The saponin assay produced stable froth persisting for more than 10 min, indicating saponin content. Additionally, the phenolic assay showed a characteristic blue coloration following Folin-Ciocalteu treatment, indicating the presence of phenolic compounds (Table 3). Overall, the extract tested positive for alkaloids, flavonoids, tannins, saponins, and phenols, indicating a rich phytochemical profile.

**Table 3:** Results of preliminary phytochemical analysis of the extract

Sl. No	Test	Results
1	Alkaloid test	Present (+)
2	Flavonoids test	Present (+)
3	Tannins test	Present (+)
4	Saponin test	Present (+)
5	Phenol test	Present (+)

Antioxidant activity of *P. dulce *extract

The antioxidant potential of *P. dulce *extract was evaluated using the DPPH radical scavenging assay. The extract exhibited a clear concentration-dependent increase in free radical scavenging activity, as evidenced by a progressive reduction in absorbance at 517 nm.

**Figure 1:**
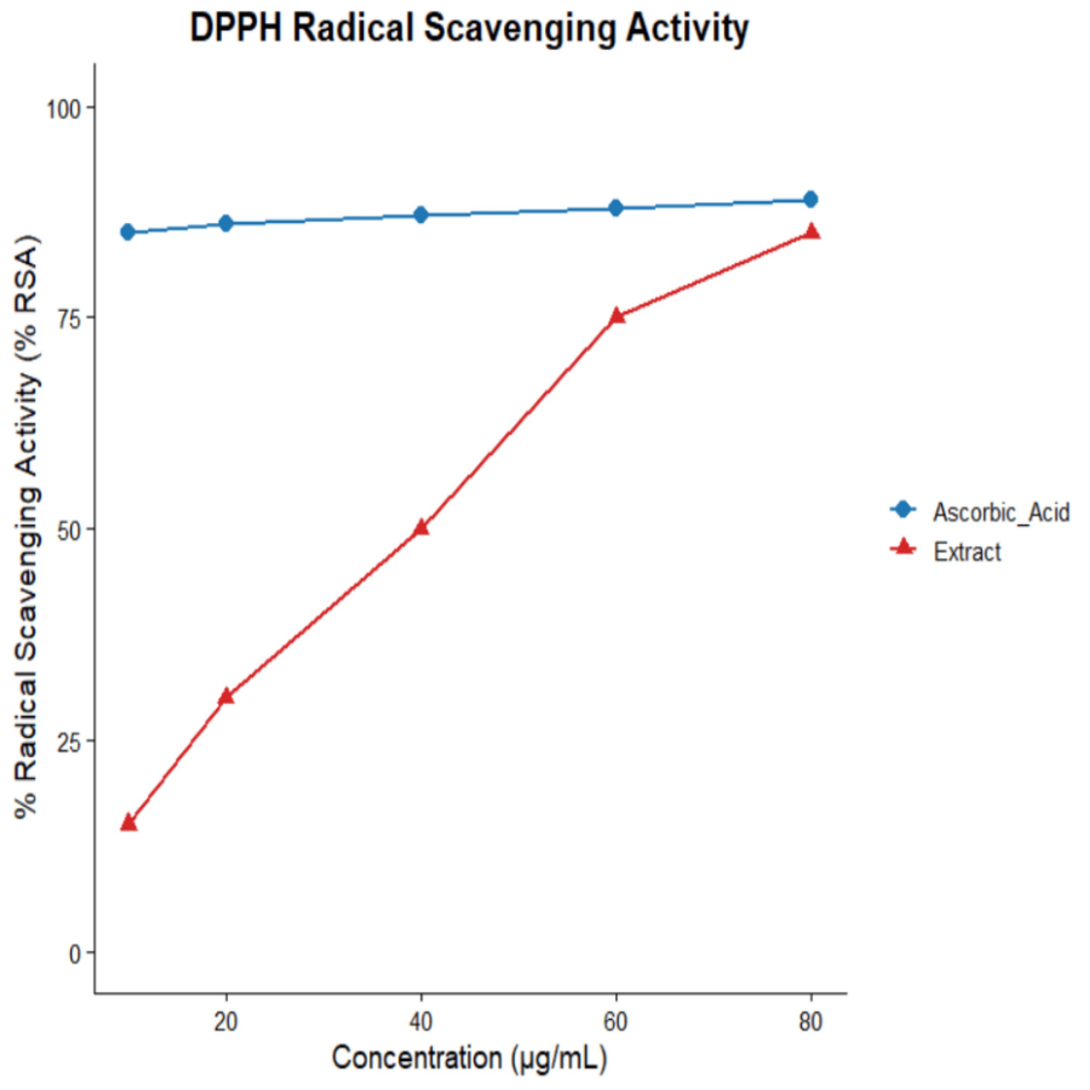
DPPH RSA of Pithecellobium dulce extract compared with ascorbic acid The x-axis represents concentration (µg/mL), and the y-axis represents percentage radical scavenging activity (%RSA). The extract demonstrated a concentration-dependent increase in radical scavenging activity, rising from ~15 ± 1.5% at 10 µg/mL to ~85 ± 3.8% at 80 µg/mL, whereas ascorbic acid exhibited consistently high activity (~85–89%). The IC₅₀ value of the extract was approximately 38 µg/mL, indicating moderate antioxidant potency. Data are presented as mean ± standard deviation of (n=3) measurements. RSA, radical scavenging activity

At lower concentrations, the extract showed moderate antioxidant activity, which increased significantly with rising concentrations. The maximum radical scavenging activity (% RSA) of the extract reached 84.83%, which was comparable to that of the standard antioxidant, ascorbic acid (~87%) (Figure 1). A steady increase in % RSA was observed across the tested concentration range, indicating enhanced hydrogen-donating capacity and effective free radical neutralization. The IC₅₀ value of the extract was approximately 38 µg/mL, suggesting moderate antioxidant potency.

GC-MS analysis of *P. dulce* extract

GC-MS analysis of the *P. dulce* fruit extract revealed a complex spectrum of phytochemical constituents, as evidenced by multiple peaks observed in the total ion chromatogram (TIC). The chromatogram demonstrated well-resolved peaks across a retention time (RT) range of approximately 6 to 50 min, indicating the presence of compounds with varying volatilities and molecular weights.

Several compounds were identified based on their RTs and mass spectral matching with standard library data. Among these, 5-hydroxymethylfurfural (HMF) was identified as the predominant compound, eluting at an RT of 9.297 min and contributing 28.08% of the total peak area with a high probability match of 92.82%. The second most abundant compound was 4H-pyran-4-one, 2,3-dihydro-3,5-dihydroxy-6-methyl, detected at an RT of 7.349 min, accounting for 13.37% of the total area.

Other major constituents included 5-(2-hydroxyethyl)-4-methylthiazole (RT 10.681 min; 7.51%), 1-isobutyl-7,7-dimethyl-octahydro-isobenzofuran-3a-ol (RT 19.948 min; 6.37%), and 1,3-dioxepane, 2-pentadecyl (RT 13.047 min; 5.29%). These compounds represent diverse chemical classes, including heterocyclic compounds, oxygenated hydrocarbons, and aliphatic derivatives.

**Figure 2:**
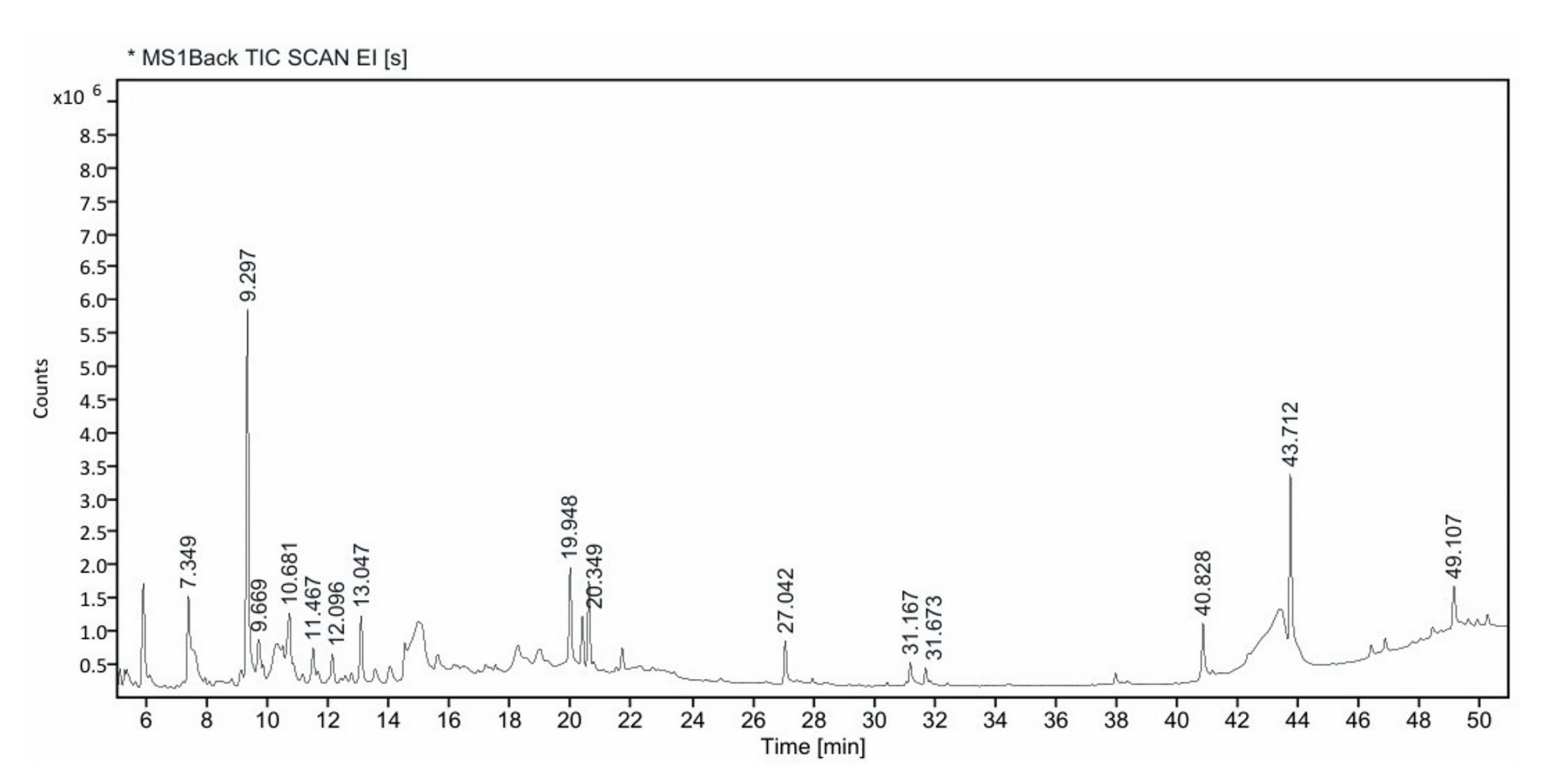
GC-MS chromatogram of Pithecellobium dulce fruit extract. The TIC shows peaks corresponding to major phytoconstituents across 6–50 min, with dominant compounds including 5-hydroxymethylfurfural (RT: 9.297 min) and 4H-pyran-4-one derivative (RT: 7.349 min). Peak areas represent relative abundance. GC-MS, gas chromatography-mass spectrometry; RT, retention time; TIC, total ion chromatogram

Additionally, minor constituents such as melezitose (RT 9.669 min; 1.97%), cyclohexanecarboxylic acid, 2-hydroxy-, ethyl ester (RT 11.467 min; 1.71%), and β-D-glucopyranose derivatives (RT 12.096 min; 1.56%) were also detected, albeit in lower concentrations (Figure 2, Table 4).

**Table 4:** Major compounds identified by GC-MS analysis of Pithecellobium dulce Area (%), relative peak area representing the proportion of each compound in the total chromatogram; GC-MS, gas chromatography-mass spectrometry; Prob (%), probability of compound identification based on NIST library matching

RT (min)	Compound Name	Area %	Prob (%)	Chemical Class
9.297	5-Hydroxymethylfurfural	28.08	92.82	Furan derivative (antioxidant)
7.349	4H-Pyran-4-one, 2,3-dihydro-3,5-dihydroxy-6-methyl	13.37	75.93	Phenolic compound
10.681	5-(2-Hydroxyethyl)-4-methylthiazole	7.51	31.97	Heterocyclic compound
19.948	1-Isobutyl-7,7-dimethyl-octahydro-isobenzofuran-3a-ol	6.37	—	Terpenoid derivative
13.047	1,3-Dioxepane, 2-pentadecyl	5.29	11.55	Lipid derivative
9.669	Melezitose	1.97	43.48	Carbohydrate
11.467	Cyclohexanecarboxylic acid, 2-hydroxy-, ethyl ester	1.71	18.36	Ester
12.096	β-D-Glucopyranose derivative	1.56	8.15	Sugar derivative

The chromatogram further exhibited several smaller peaks at higher RTs (27-49 min), suggesting the presence of less abundant or higher molecular weight phytoconstituents that may contribute synergistically to the overall biological activity of the extract.

Overall, the GC-MS analysis indicates that *P. dulce *fruit extract is rich in furfural derivatives, pyranone compounds, thiazole derivatives, and carbohydrate-related molecules, suggesting potential relevance of its chemically diverse nature and potential for pharmacological applications.

Toxicological assessment and dose selection

**Figure 3:**
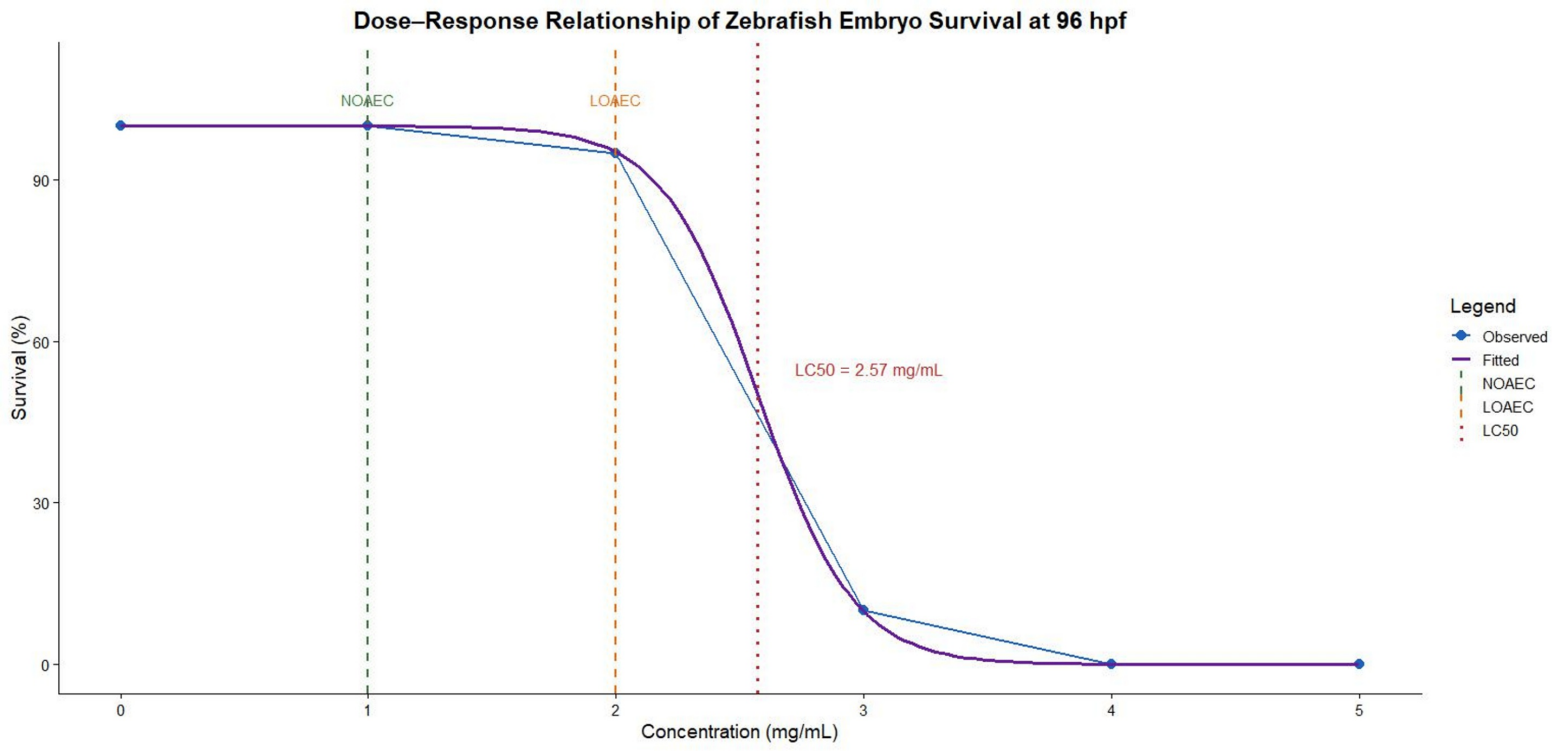
Dose–response survival curve of zebrafish embryos exposed to Pithecellobium dulce extract at 96 hours post-fertilization (hpf) Survival (%) was assessed in zebrafish embryos (n = 20 per group) exposed to increasing concentrations of *P. dulce *extract. A concentration-dependent decline in survival was observed, with 100% survival at 1 mg/mL, 95% at 2 mg/mL, and complete mortality at ≥4 mg/mL. Observed data (blue) and fitted dose–response curve (purple; binomial GLM) are shown. NOAEC (1 mg/mL) and LOAEC (2 mg/mL) are indicated by dashed green and orange lines, respectively. The LC_50_ (2.57 mg/mL; 95% CI: 2.34–2.81 mg/mL) is marked by a red dotted line. GLM, generalized linear model; LOAEC, lowest observed adverse effect concentration; NOAEC, no observed adverse effect concentration

A concentration-dependent increase in mortality was observed, with no mortality at 1 mg/mL and minimal mortality (5%) at 2 mg/mL (Table 5), while a sharp decline in survival occurred at higher concentrations, reaching 90% mortality at 3 mg/mL and complete mortality at ≥4 mg/mL by 96 hpf. The NOAEC was identified at 1 mg/mL, whereas the LOAEC was determined at 2 mg/mL. The LC_50_ at 96 hpf was calculated to be 2.573 mg/mL (95% CI: 2.340-2.806 mg/mL), indicating a steep concentration-dependent toxicity between 2 and 3 mg/mL. (Figure 3). These estimates are based on quantal mortality data and should be interpreted within the constraints of the experimental design.

**Table 5:** Dose-dependent effects of Pithecellobium dulce extract on survival, hatching, and morphological development in zebrafish embryos (24–96 hpf) Zebrafish embryos (n = 20 per group) were exposed to increasing concentrations of *P. dulce *extract and monitored at 24, 48, 72, and 96 hours post-fertilization (hpf). Parameters assessed included mortality, hatching rate, morphological abnormalities (edema, hemorrhage, tail detachment), and cardiac activity.

Concentration (mg/mL)	Time (hpf)	Total (n)	Dead (n)	Mortality (%)	Hatching	Morphological Abnormalities	Cardiac Activity
1	24	20	0	0	Normal	None	Normal
1	48	20	0	0	Normal	None	Normal
1	72	20	0	0	Normal	None	Normal
1	96	20	0	0	Normal	None	Normal
2	24	20	0	0	Normal	None	Normal
2	48	20	1	5	Normal	None	Normal
2	72	20	1	5	Normal	None	Normal
2	96	20	1	5	Normal	None	Normal
3	24	20	8	40	Delayed	Minimal/none in survivors	Present (reduced survival)
3	48	20	14	70	Delayed	Minimal/none in survivors	Present (in viable embryos)
3	72	20	17	85	Delayed	Minimal/none in survivors	Present (in viable embryos)
3	96	20	18	90	Delayed	Minimal/none in survivors	Present (in viable embryos)
4	24	20	16	80	Delayed/absent	Edema, hemorrhage, tail not detached	Reduced/absent
4	48	20	19	95	Absent	Severe abnormalities	Mostly absent
4	72	20	20	100	Absent	Severe abnormalities	Absent
4	96	20	20	100	Absent	Severe abnormalities	Absent
5	24	20	19	95	Absent	Severe abnormalities	Absent
5	48	20	20	100	Absent	Severe abnormalities	Absent
5	72	20	20	100	Absent	Severe abnormalities	Absent
5	96	20	20	100	Absent	Severe abnormalities	Absent

Based on these findings, a lower concentration of 1 mg/mL was selected for subsequent behavioral and molecular analyses to ensure safety during prolonged exposure as it provided an optimal balance between safety and biological activity.

Behavioral assessment of zebrafish larvae

**Figure 4:**
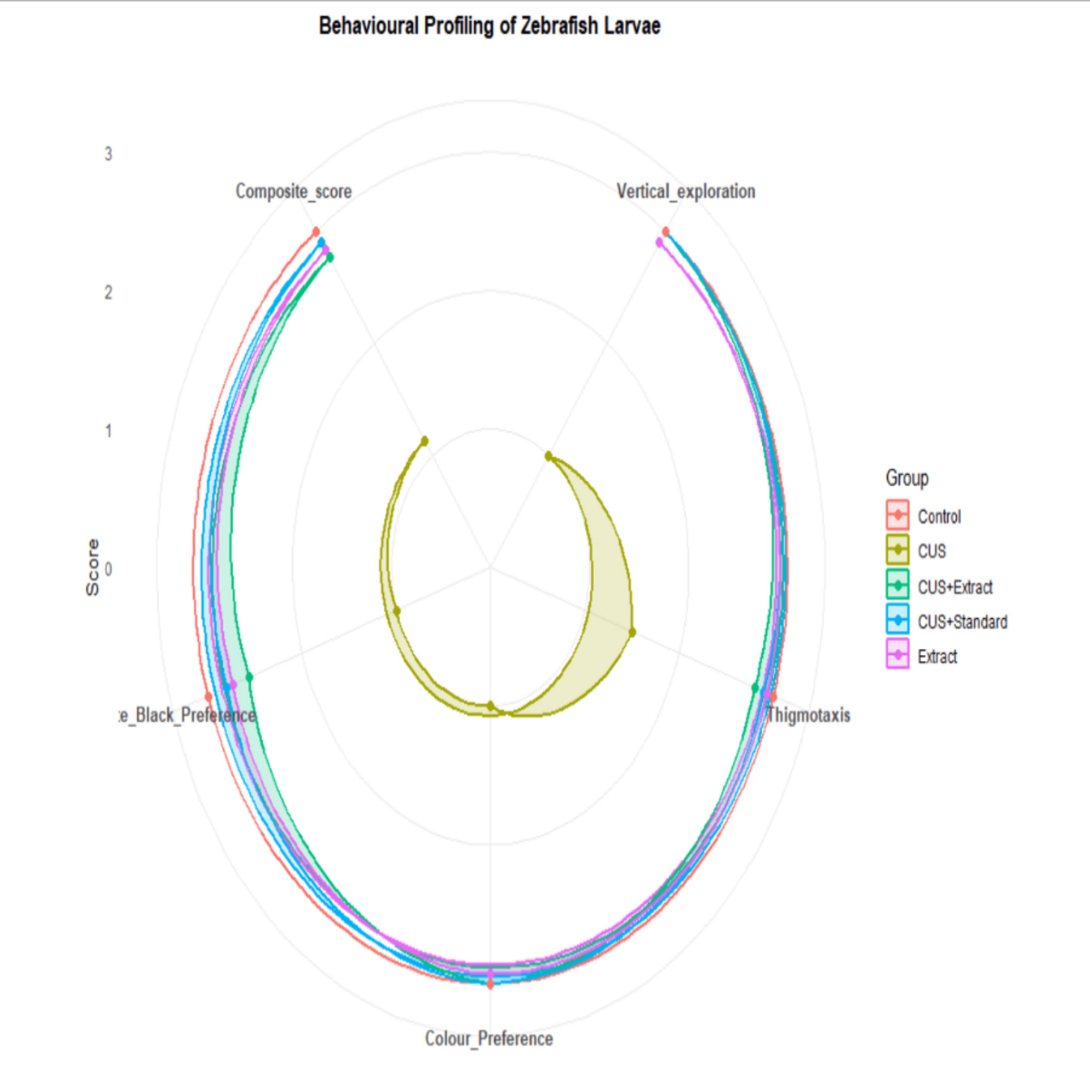
Behavioural profiling of zebrafish larvae using radar plot analysis Radar plot illustrating behavioral parameters including vertical exploration, thigmotaxis, color preference, light–dark (black–white) preference, and composite score across experimental groups. The CUS group exhibited a marked reduction in all behavioral parameters, indicating stress-induced behavioral deficits. The standard drug used was sertraline hydrochloride, and scores were similar to controls. Treatment with standard drug and *P. dulce* extract improved behavioral performance, with values approaching control levels. Data represent mean scores for each group. CUS, chronic unpredictable stress

**Figure 5:**
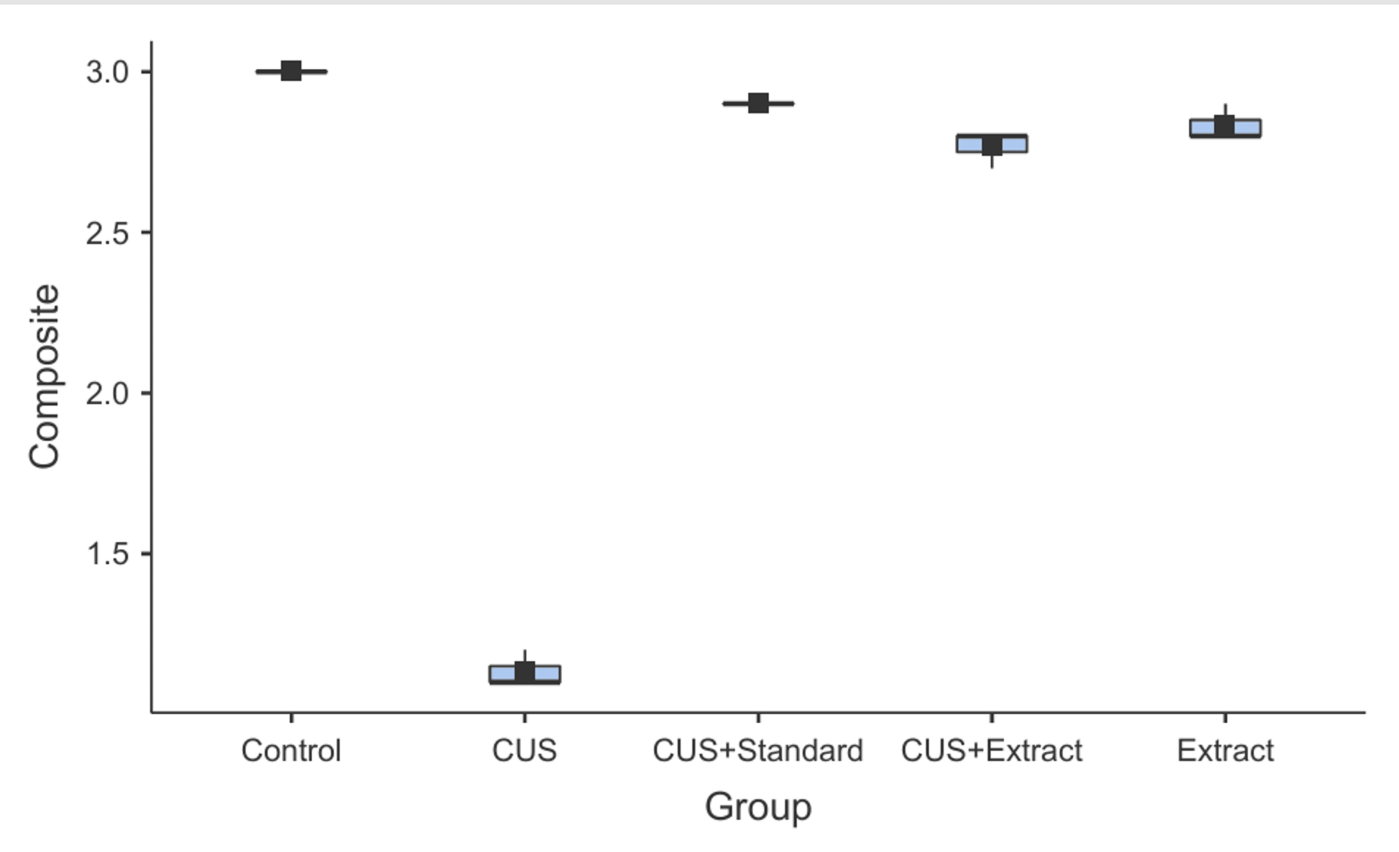
Comparison of composite behavioural scores among experimental groups Data are presented as mean ± SD of three independent experimental replicates (n = 3), with each replicate consisting of 20 larvae per group. The CUS group showed a significant reduction in behavioral score compared to controls, while treatment with *P. dulce *extract and the standard drug sertraline hydrochloride significantly improved behavioral outcomes. Statistical analysis was performed using one-way ANOVA (F(4,10) = 921, p < 0.001, η² = 0.997) followed by Tukey’s post hoc test. CUS, chronic unpredictable stress

Behavioral analysis of zebrafish larvae was associated with pronounced alterations across experimental groups, as illustrated by radar plot and boxplot representations (Figures 4, 5). Analysis of composite behavioral scores using one-way ANOVA demonstrated a highly significant difference among groups (F(4,10) = 921, p < 0.001, η² = 0.997), indicating clear separation between experimental conditions. The high effect size reflects low within-group variability due to the use of replicate means (n = 3), which reduces intra-group noise.

Individual behavioral parameters were further evaluated using the Kruskal-Wallis test, revealing significant differences among groups for vertical exploration (χ²(4) = 12.2, p = 0.0156), thigmotaxis (χ²(4) = 10.7, p = 0.0305), color preference (χ²(4) = 12.0, p = 0.0174), and scototaxis (χ²(4) = 12.9, p = 0.0118). Effect size estimates indicated large effects across all parameters (η²[H] = 0.667-0.890), reflecting substantial behavioral divergence between groups.

Post hoc analysis using Dunn’s test with Bonferroni correction demonstrated that the CUS group differed significantly from the control group in thigmotaxis (p = 0.0284), color preference (p = 0.0468), and scototaxis (p = 0.00855), confirming successful induction of stress-associated behavioral deficits. Significant differences were also observed between the CUS group and treatment groups (CUS + extract and CUS + standard) in color preference (p = 0.0468), indicating partial behavioral recovery. Other pairwise comparisons did not reach statistical significance following correction, likely reflecting the limited number of independent replicates.

The control group consistently exhibited high behavioral scores across all parameters, indicative of normal exploratory behavior and low anxiety levels (Figure 4). In contrast, the CUS group showed a marked reduction across all behavioral endpoints. Tukey’s post hoc test following ANOVA indicated that composite behavioral scores were significantly reduced in the CUS group compared to the control group (p < 0.001), validating effective stress induction.

Data are presented as mean ± standard deviation (SD) of three independent biological replicates (n = 3), with each replicate comprising 20 larvae. Treatment with the standard drug (sertraline hydrochloride) significantly restored behavioral performance, with scores comparable to the control group (p > 0.05), indicating effective reversal of stress-induced deficits.

Similarly, the *P. dulce* extract-treated group (CUS + extract) demonstrated significant improvement in behavioral performance relative to the untreated CUS group (p < 0.001). However, scores remained modestly lower than the control group (p < 0.05), suggesting partial yet meaningful recovery. The extract-alone group exhibited behavioral profiles comparable to the control group (p > 0.05), indicating the absence of adverse behavioral effects.

The boxplot representation of composite behavioral scores (Figure 5) further supports these findings by illustrating distribution patterns and variability within each group. The CUS group displayed a reduced median score relative to controls, whereas both treatment groups (CUS + standard and CUS + extract) showed a clear upward shift toward higher scores, consistent with behavioral recovery. Collectively, these results demonstrate that CUS induces significant behavioral impairment, while treatment with sertraline and *P. dulce *extract effectively ameliorates these deficits.

BDNF and TPH2 gene expression profiling

**Figure 6:**
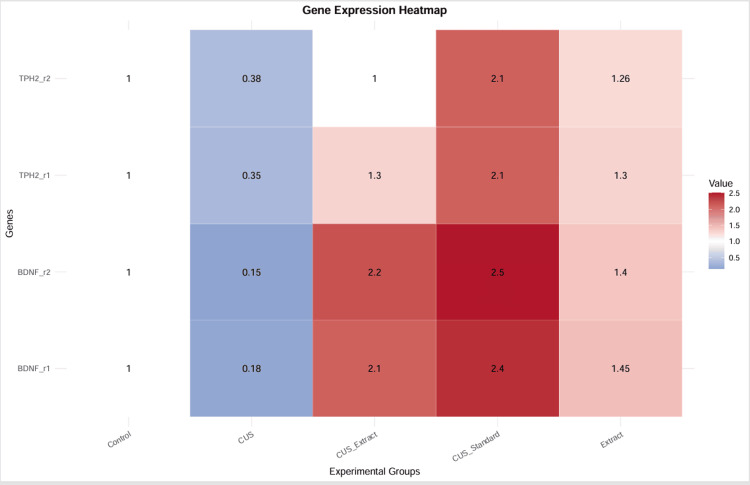
Heatmap representing relative gene expression levels of BDNF and TPH2 Heatmap representing relative gene expression levels of BDNF and TPH2 (r1 and r2 replicates) across experimental groups (control, CUS, CUS + extract, CUS + standard, and extract). Expression values are presented as fold change relative to the control group (normalized to 1). The color gradient ranges from blue (downregulation) to red (upregulation), indicating relative expression levels. The CUS group shows marked downregulation of both BDNF and TPH2, whereas treatment with the standard drug (sertraline hydrochloride) shows a pronounced upregulation of gene expression. The *P. dulce* extract-treated group (CUS + extract) also demonstrates restoration of gene expression compared to the CUS group, although to a lesser extent than the standard treatment. The extract-alone group exhibits mild upregulation compared to controls, suggesting a potential modulatory effect. These observations are based on pooled samples with duplicate measurements and are presented as indicative trends. BDNF, brain-derived neurotrophic factor; CUS, chronic unpredictable stress; TPH2, tryptophan hydroxylase-2

Gene expression analysis of BDNF and TPH2 was associated with marked alterations across experimental groups (Figure 6). Data are presented as mean ± standard deviation (SD) of duplicate technical measurements (n = 2). In the CUS group, both genes showed substantial downregulation compared to controls, with BDNF expression reduced to 0.17 ± 0.02-fold and TPH2 to 0.37 ± 0.02-fold, indicating stress-induced molecular suppression.

Treatment with *P. dulce* extract (CUS + Extract) resulted in a notable recovery in gene expression relative to the CUS group. BDNF expression increased to approximately 1.26- to 1.45-fold, approaching near-control levels, while TPH2 expression also improved (~1.3- to 1.4-fold), suggesting a modulatory effect on neurotrophic and serotonergic pathways.

The standard drug, sertraline hydrochloride, produced the highest upregulation, with BDNF reaching 2.45 ± 0.07-fold and TPH2 approximately 2.1-fold, exceeding control levels. The extract-alone group exhibited mild elevation in BDNF (1.43 ± 0.04-fold) and TPH2 (1.28 ± 0.03-fold) compared to controls, indicating no adverse molecular effects.

Due to the use of pooled samples with duplicate technical measurements (n = 2) and the absence of independent biological replicates, statistical significance testing was not performed. Therefore, the results are presented as indicative trends rather than inferential conclusions.

## Discussion

The present study demonstrates that CUS induces significant behavioral alterations in zebrafish larvae, including reduced exploratory behavior, impaired vertical movement, and increased preference for darker environments. These behavioral changes are indicative of anxiety- and depression-like phenotypes and are consistent with previous findings in zebrafish stress models [27,28]. The statistically significant differences observed across behavioral parameters further support the robustness of the stress-induced phenotype.

The observed reduction in vertical exploration and restricted movement in CUS-exposed larvae may reflect a state of behavioral despair, characterized by diminished motivation and impaired stress coping. Zebrafish models have been shown to capture such despair-like phenotypes, including reduced locomotion and increased stress sensitivity [14]. Furthermore, stress exposure has been associated with neuroendocrine dysregulation and altered neuronal activity, supporting the biological relevance of the observed behavioral impairments.

Zebrafish are increasingly recognized as a useful model for studying neuropsychiatric disorders due to their conserved neurochemical pathways and sensitivity to pharmacological interventions. Previous studies have demonstrated that zebrafish exhibit behavioral and physiological responses analogous to those observed in mammalian systems, supporting their application in antidepressant screening [13,15]. However, the translational relevance of findings from zebrafish larvae to higher vertebrate systems remains limited and requires further validation.

An additional behavioral dimension explored in this study was color preference, which provided insight into affective state alterations. The increased preference for darker environments observed in the CUS group suggests heightened anxiety-like behavior, whereas partial normalization of color preference following *P. dulce* treatment suggests improvement in emotional behavior. Environmental color has been shown to influence zebrafish behavior and physiological responses, including stress-related outcomes [25].

Treatment with *P. dulce* extract resulted in a partial but meaningful recovery of behavioral deficits, while the standard drug sertraline produced near-complete restoration. The observed improvements were statistically significant and supported by large effect sizes. However, the magnitude of the effect may partially reflect reduced variability due to the scoring system rather than purely biological differences. Natural compounds have been reported to mitigate stress-induced behavioral alterations through modulation of neurochemical pathways, including serotonergic and GABAergic systems [16]. However, these mechanisms remain inferential in the present study and are not directly validated.

The selection of 1 mg/mL as the working concentration was guided by toxicological findings, which identified this dose as the NOAEC. This ensured safety during prolonged exposure while maintaining biological activity. The steep increase in mortality observed between 2 and 3 mg/mL further justified the use of a conservative dose, indicating a narrow transition between safe and toxic concentrations.

At the molecular level, the antidepressant-like effects of *P. dulce* may be associated with modulation of neuroplasticity and serotonergic signaling pathways. Impaired BDNF signaling is a hallmark of depression and is associated with reduced synaptic plasticity and neuronal survival [6]. Similarly, serotonergic dysfunction plays a central role in depression, with TPH2 acting as the rate-limiting enzyme in serotonin synthesis [5]. The observed changes in BDNF and TPH2 mRNA expression suggest a potential involvement of these pathways; however, these findings are based on gene expression data alone and lack protein-level validation or direct measurement of neurotransmitter levels. Therefore, these interpretations should be considered associative rather than definitive.

The antioxidant activity demonstrated by *P. dulce* extract in the DPPH assay further supports its potential biological relevance. Oxidative stress is increasingly recognized as a contributor to depression pathophysiology, leading to cellular damage and neuronal dysfunction [29]. Bioactive compounds such as 5-HMF, identified in the GC-MS analysis, have been reported to exhibit neuroprotective and antioxidant properties [30]. However, compound identification in the present study was based on library matching, and some identifications should be considered tentative. Additionally, the relationship between phytochemical constituents and observed biological effects remains correlational.

Collectively, the findings of this study suggest that *P. dulce* extract exhibits antidepressant-like effects in a zebrafish larvae model, potentially involving modulation of oxidative stress, neuroplasticity, and serotonergic pathways. However, these effects are based on indirect evidence derived from behavioral, molecular, and phytochemical analyses, and should be interpreted cautiously.

Despite these findings, several limitations should be acknowledged. Behavioral assessment using a semi-quantitative scoring system, although blinded, may introduce observer-related variability, and inter-rater reliability was not assessed. Gene expression analysis was performed using pooled samples with limited replication and was not fully MIQE-compliant. Additionally, extract standardization and validation of GC-MS-identified compounds were not performed. Cortisol levels and pharmacokinetic parameters were not assessed, limiting confirmation of stress induction and translational interpretation. The sample size was based on standard practices without formal power calculation. Future studies should incorporate automated behavioral tracking, molecular validation, and pharmacokinetic analysis, and extract standardization to strengthen mechanistic and translational relevance.

Altogether, these findings provide preliminary evidence supporting the potential neurobehavioral effects of *P. dulce *extract; however, mechanistic validation and quantitative behavioral and molecular analyses are required to substantiate these observations.

## Conclusions

In summary, the present study demonstrates that CUS induces measurable behavioral alterations in zebrafish larvae, as assessed through exploratory activity, vertical movement, and color preference, and that treatment with *P. dulce *extract is associated with significant improvement in these stress-induced changes, suggesting a potential modulatory effect on affective responses. These effects may be linked to alterations in neuroplasticity- and serotonergic-related markers (BDNF and TPH2), along with antioxidant activity observed in vitro; however, these interpretations are based on indirect evidence from gene expression and phytochemical analyses and should be considered associative rather than definitive. The presence of diverse bioactive phytochemicals in *P. dulce* may contribute to the observed effects, although compound identification was based on library matching without validation using reference standards and without quantitative standardization, and mechanistic pathways were not directly investigated. While the zebrafish larvae model provides a useful platform for studying stress-related behavioral changes, the translational relevance of these findings remains limited in the absence of pharmacokinetic evaluation and validation in higher vertebrate models. Additionally, methodological limitations, including semi-quantitative behavioral scoring, lack of inter-rater reliability assessment, pooled sampling for gene expression analysis, and incomplete MIQE-compliant qPCR validation, may influence interpretation. Overall, the findings provide preliminary but consistent evidence supporting the potential of *P. dulce* as a candidate for modulating stress-associated behavioral and molecular alterations, warranting further studies involving mechanistic validation, protein-level analysis, neurotransmitter quantification, pharmacokinetic profiling, and extract standardization to establish broader relevance.
